# A High Throughput Screen Identifies Potent and Selective Inhibitors to Human Epithelial 15-Lipoxygenase-2

**DOI:** 10.1371/journal.pone.0104094

**Published:** 2014-08-11

**Authors:** J. Brian Jameson, Auric Kantz, Lena Schultz, Chakrapani Kalyanaraman, Matthew P. Jacobson, David J. Maloney, Ajit Jadhav, Anton Simeonov, Theodore R. Holman

**Affiliations:** 1 Chemistry and Biochemistry Department, University of California Santa Cruz, Santa Cruz, California, United States of America; 2 National Center for Advancing Translational Sciences, National Institutes of Health, Bethesda, Maryland, United States of America; 3 Department of Pharmaceutical Chemistry, School of Pharmacy, University of California San Francisco, San Francisco, California, United States of America; Albert-Ludwigs-University, Germany

## Abstract

Lipoxygenase (LOX) enzymes catalyze the hydroperoxidation of arachidonic acid and other polyunsaturated fatty acids to hydroxyeicosatetraenoic acids with varying positional specificity to yield important biological signaling molecules. Human epithelial 15­lipoxygenase­2 (15-LOX-2) is a highly specific LOX isozyme that is expressed in epithelial tissue and whose activity has been correlated with suppression of tumor growth in prostate and other epithelial derived cancers. Despite the potential utility of an inhibitor to probe the specific role of 15-LOX-2 in tumor progression, no such potent/specific 15­LOX­2 inhibitors have been reported to date. This study employs high throughput screening to identify two novel, specific 15­LOX­2 inhibitors. MLS000545091 is a mixed-type inhibitor of 15-LOX-2 with a K_i_ of 0.9+/−0.4 µM and has a 20-fold selectivity over 5-LOX, 12-LOX, 15-LOX-1, COX-1, and COX-2. MLS000536924 is a competitive inhibitor with a K_i_ of 2.5+/−0.5 µM and also possesses 20-fold selectivity toward 15-LOX-2 over the other oxygenases, listed above. Finally, neither compound possesses reductive activity towards the active-site ferrous ion.

## Introduction

Lipoxygenase (LOX) enzymes catalyze the hydroperoxidation of polyunsaturated fatty acids with varying levels of substrate preference and positional specificity. Human LOX enzymes are named for the primary position of hydroperoxide installation on arachidonic acid (AA), with two human LOX isozymes hydroperoxidating at carbon-15 of AA. Both reticulocyte 15-LOX-1 (15-LOX-1 or 12/15-LOX) and epithelial 15-LOX-2 (15-LOX-2) appear to have distinct and important roles in cancer progression but current data are highly conflicted as to whether their enzymatic activities are beneficial or deleterious [1]. 15-LOX-2 is expressed in skin, cornea, prostate, and lung [2]. In humans, expression level of 15-LOX-2 is inversely related to prostate tumor volume [3]. In mice, however, overexpression of 15-LOX-2 leads to hyperplasia [4]. 15-LOX-1 is primarily expressed in reticulocytes, eosinophils, and macrophages [1]. In certain cancers, the expression of 15-LOX-1 is increased in malignant tissue; however, in other cases its expression correlates with suppression of tumor growth [5]. While the exact mechanism by which 15-LOX-1 and 15-LOX-2 elicit their effects is unknown, there are data that suggest the differences in their biological activities are evoked by differences in the substrate preferences of the two enzymes [6]. It has also been speculated that 15-LOX-2 may have some biological role independent of its enzymatic function, as seen with splice variants that lack enzymatic activity [7].

**Figure 1:**
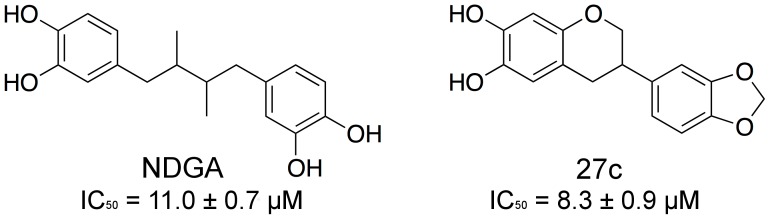
Current known 15-LOX-2 inhibitors (IC_50_ values are in µM).

Given the complex biological role of both 15-LOX-1 and 15-LOX-2, it would be helpful to have specific inhibitors of these two isozymes to better delineate their roles in human disease. Potent and selective inhibitors of 15-LOX-1 have been identified [8]–[12], however, no potent and selective inhibitors for 15-LOX-2 have been observed. There are currently two inhibitors with potency against 15-LOX-2 in the literature (Figure 1). The first, nordihydroguaiaretic acid (NDGA), is a redox active LOX inhibitor and has an IC_50_ value of 11.0+/−0.7 µM for 15-LOX-2 [13]. The second is the flavanoid based compound, 27c, which has an IC_50_ value of 8.3+/−0.9 µM [13]. However, neither of these compounds is selective toward 15-LOX-2. Due to this dearth of potent and selective 15-LOX-2 inhibitors, we set out to identify novel inhibitors against 15-LOX-2 using our previously reported high throughput screen [14] that has already yielded potent and selective compounds against 15-LOX-1 [8] and platelet 12-LOX (12-LOX) [15], [16]. In the current work, we report two potent and selective 15-LOX-2 inhibitors that were characterized by kinetic analysis and could be used further to characterize the biological mechanism by which 15-LOX-2 affects disease progression.

## Materials and Methods

### Materials

All commercial fatty acids were purchased from Nu Chek Prep, Inc. (MN, USA) and were further re-purified using a Higgins HAISIL column (5 µm, 250×10 mm) C-18 column. An isocratic elution of 85% A (99.9% methanol and 0.1% acetic acid): 15% B (99.9% water and 0.1% acetic acid) was used to purify all the fatty acids. Post purification, the fatty acids were stored at −80°C for a maximum of 6 months. BWb70c and NDGA were purchased from Sigma/Aldrich Chemicals. The inhibitors were obtained from the NIH Molecular Libraries Small Molecule Repository (MLSMR): (https://mli.nih.gov/mli/compound-repository/). All other chemicals were reagent grade or better and were used without further purification.

### Overexpression and Purification of Lipoxygenases

Different lipoxygenases, such as 15-LOX-1, 15-LOX-2, and 12-LOX were expressed as N-terminal His6-tagged proteins and were purified via immobilized metal affinity chromatography (IMAC) using an Ni-NTA resin for 15-LOX-1 [12], and 15-LOX-2 [17], and a Ni-IDA resin for 12-LOX [18]. The protein purity was evaluated by SDS-PAGE analysis and was found to be greater than 90%. Human leukocyte 5-lipoxygenase (5-LOX) was expressed as a non-tagged protein and used as a crude ammonium sulfate protein fraction, as published previously [19], [20].

### High-throughput Screen Materials

Dimethyl sulfoxide (DMSO) ACS grade was from Fisher, while ferrous ammonium sulfate, Xylenol Orange (XO), sulfuric acid, and Triton X-100 were obtained from Sigma-Aldrich.

### 15-Lipoxygenase-2 qHTS Assay

All screening operations were performed on a fully integrated robotic system (Kalypsys Inc, San Diego, CA) as described elsewhere [21]. Three µL of enzyme (approximately 50 nM 15-LOX-2, final concentration) was dispensed into 1536-well Greiner black clear-bottom assay plates. Compounds and controls (23 nL) were transferred via Kalypsys PinTool, equipped with 1536-pin array, at concentrations ranging from 57 µM to 0.73 nM. The plate was incubated for 15 min at room temperature, and then a 1 µL aliquot of substrate solution (50 µM arachidonic acid final concentration) was added to start the reaction. The reaction was stopped after 6.5 min by the addition of 4 µL FeXO solution (final concentrations of 200 µM XO and 300 µM ferrous ammonium sulfate in 50 mM sulfuric acid). After a short spin (1000 rpm, 15 sec), the assay plate was incubated at room temperature for 30 minutes. The absorbances at 405 and 573 nm were recorded using ViewLux high throughput CCD imager (Perkin-Elmer, Waltham, MA) using standard absorbance protocol settings. During dispense, enzyme and substrate bottles were kept submerged into a +4°C recirculating chiller bath to minimize degradation. Plates containing DMSO only (instead of compound solutions) were included approximately every 50 plates throughout the screen to monitor any systematic trend in the assay signal associated with reagent dispenser variation or decrease in enzyme specific activity. Data were normalized to controls, and plate-based data corrections were applied to filter out background noise. Average Z′ was 0.69 across 688 1536-well assay plates. Signal to background was 3.4. The qHTS yielded 1,562,952 activity points in dose response format. We had identified 163 chemical clusters and 191 singletons using the cheminformatics analysis process previously described [21].

### Lipoxygenase UV-Vis Assay

The inhibitor compounds were screened initially using one concentration point at 25 µM on a Perkin-Elmer Lambda 40 UV/Vis spectrophotometer. The percent inhibition was determined by comparing the enzyme rates of the control (DMSO solvent) and the inhibitor sample by following the formation of the conjugated diene product at 234 nm (ε = 25,000 M^−1^ cm^−1^). The reactions were initiated by adding 200 nM 15-LOX-2, 30 nM 12-LOX, 40 nM 15-LOX-1, or approximately 100–300 nM (5–10 µL) of 5-LOX crude extract to a cuvette with 2 mL reaction buffer, constantly stirred using a magnetic stir bar at room temperature (22°C). Reaction buffers used for various LOX isozymes were as follows: 25 mM HEPES (pH 7.3), 0.3 mM CaCl_2_, 0.1 mM EDTA, 0.2 mM ATP, 0.01% Triton X-100, 10 µM AA for the crude, ammonium sulfate precipitated 5-LOX; and 25 mM HEPES (pH 7.5), 0.01% Triton X-100, 10 µM AA for 15-LOX-2, 15-LOX-1, and 12-LOX. The substrate concentration was quantitatively determined by allowing the enzymatic reaction to go to completion in the presence of 15-LOX-2. For the inhibitors that showed more than 50% inhibition in the one-point screen, IC_50_ values were obtained by determining the % inhibition, relative to solvent vehicle only, at various inhibitor concentrations. The data were then plotted against inhibitor concentration, followed by a hyperbolic saturation curve fit (assuming total enzyme concentration [E]≪IC_50_). It should be noted that all of the potent inhibitors displayed greater than 80% maximal inhibition, unless otherwise stated in the tables. All inhibitors were stored at −20°C in DMSO. NDGA was utilized as positive control for the selectivity assays for the various LOX isozymes.

### Steady State Inhibition Kinetics

The steady-state kinetics experiments were performed with MLS000545091 and MLS000536924 to determine the mode of inhibition. Inhibitor concentrations of 0, 1, 2 and 5 µM were used. Reactions were initiated by adding approximately 200 nM of 15-LOX-2 to a constantly stirring 2 mL cuvette containing 25 mM HEPES buffer (pH 7.5) and 1–40 µM AA, in the presence of 0.01% Triton X-100. LOX reaction rates were determined by monitoring the formation of the conjugated product, 15-HPETE, at 234 nm (ε = 25 000 M^−1^ cm^−1^) with a Perkin-Elmer Lambda 40 UV/Vis spectrophotometer. The substrate concentration was quantitatively determined by allowing the enzymatic reaction to proceed to completion. Initial enzymatic rates were plotted versus substrate concentration at various inhibitor concentrations, and subsequently fitted to the Henri–Michaelis–Menten equation, using KaleidaGraph (Synergy) to determine the microscopic rate constants, *V*_max_ (µmol/min/mg) and *V*_max_/*K*_M_ (µmol/min/mg/µM). *K*_M_/*V*_max_ was replotted versus inhibitor concentration to yield K_i_ and 1/*V*_max_ was replotted versus inhibitor concentration to yield K_i_′. K_i_ and K_i_′ are defined as the equilibrium constants of dissociation from the enzyme and enzyme substrate complex, respectively

### Pseudoperoxidase Assay

The pseudo-peroxidase activity was determined with 15-LOX-2 enzyme, using BWb70c as a positive control, and 13-HPODE as the oxidizing product, on a Perkin-Elmer Lambda 40 UV/Vis spectrophotometer, as described previously [22]. Activity was determined by monitoring the decrease at 234 nm (product degradation) in buffer (50 mM Sodium Phosphate (pH 7.4), 0.3 mM CaCl_2_, 0.1 mM EDTA, 0.01% Triton ×100, and 20 µM 13-HPODE). About 400 nM 15-LOX-2 was added to 2 mL buffer containing 20 µM 13-HPODE, constantly mixed with a rotating stir bar (22°C). Reaction was initiated by addition of 20 µM inhibitor (1∶1 ratio to product). The percent consumption of 13-HPODE was recorded and loss of product less than 20% was not considered as viable redox activity. Individual controls were conducted consisting of enzyme alone with product and MLS000545091 and MLS000536924 alone with enzyme. These negative controls formed the baseline for the assay, reflecting non-pseudo-peroxidase dependent hydroperoxide product decomposition. To rule out the auto-inactivation of the enzyme from pseudo-peroxidase cycling, the 15-LOX-2 residual activity was measured after the assay was complete: 20 µM AA was added to the reaction mixture and the residual activity was determined by comparing the initial rates with inhibitor and 13-HPODE versus inhibitor alone, since the inhibitor by itself inherently lowers the rate of the oxygenation. Activity is characterized by direct measurement of the product formation with the increase of absorbance at 234 nm.

### Cyclooxygenase assay

Cyclooxygenase activity assay was performed as previously described [8]. Approximately 3 µg of either ovine COX-1 (COX-1) or human recombinant COX-2 (COX-2) (Cayman Chemical) were added to buffer containing 0.1 M Tris-HCl buffer (pH 8.0), 5 mM EDTA, 2 mM phenol and 1 µM hematin at 37°C. The selected inhibitors were added to the reaction cell, followed by an incubation of 5 minutes with either of the COX enzymes. The reaction was then initiated by adding 100 µM AA in the reaction cell, as indicated in enzymatic protocol (Cayman Chemicals). Data were collected using a Hansatech DW1 oxygen electrode and the consumption of oxygen was recorded. Indomethacin and the solvent (DMSO), were used as positive and negative controls, respectively, and the percent inhibition of the enzyme was calculated by comparing the rates of the samples to the controls.

### HPLC inhibitor Modification Assay

Compounds were confirmed to be un-modified by the enzyme. Both 15-LOX-2 inhibitors (10 µM) were incubated with the enzyme in the presence and absence of 10 µM AA. The mixture was extracted and analyzed via HPLC using a C18 reverse phase column with a gradient of 50–100% acetonitrile versus water (0.1% TFA). Retention times and absorbance spectra were compared to enzyme-free control reaction.

### Computational Docking of Inhibitors to 15-LOX-2

The 15-LOX-2 inhibitors MLS000545091 and MLS000536924 were docked to the crystal structure of 15-LOX-2 (PDB ID: 4NRE) [23]. The competitive inhibitor found in the 15-LOX-2 structure is a substrate mimic with a long aliphatic chain that binds in the active site. In order to successfully dock our much larger inhibitors in the active site, we found it necessary to treat the active site as conformationally flexible. Specifically, we docked MLS000536924 using the InducedFit Docking (IFD) software (Schrodinger Inc). IFD uses a combination of rigid docking (Glide) and protein side chain optimization (PRIME) to dock the ligand in the binding site [24]. Prior to using IFD, the protein structure was subjected to a protein preparation step. During this step solvent and other unwanted ligands, except the metal ion (Fe^3+^) and the active site inhibitor, were removed from the structure, hydrogen atoms were added and the binding site was energy-minimized, such that the heavy atoms of the protein did not move beyond 0.3 Å from their initial position. Default parameters were used during IFD.

After the induced fit docking, most active site residues remained largely unchanged, but the side chains of residues Leu 420 and Leu 610 moved slightly (heavy atom root mean square difference (RMSD) between before and after IFD is 1.4 Å) to accommodate the aromatic ring of the ligand. The resulting MLS000536924 bound structure was then used in the subsequent standard Glide docking protocol. In addition to the 15-LOX-2 inhibitors of this work, the selective 15-LOX-1 ligands [8], [25] (ML094 and ML351) and selective 12-LOX inhibitors [15], [16] (ML127 and ML355) were docked as control molecules, since they are known not to inhibit 15-LOX-2. Glide (version 5.8515) docking was performed using the extra precision (XP) scoring function. In our earlier work, the force-field based rescoring (MM-GBSA) of docking poses was shown to yield better rank-ordering of ligands [26]. Therefore, after the Glide docking, the docking poses were rescored using MM-GBSA. During MM-GBSA rescoring, the ligand was minimized in the binding pocket, while the protein was held rigid. After ligand minimization, the protein and the ligand were separated from the complex and their energies were evaluated. All energy evaluations were performed using the OPLS-all atom force-field, with a generalized Born implicit solvent energy function. A relative binding energy was calculated for each ligand by subtracting energies of the ligand and the protein from the complex. The relative binding energy was used for rank ordering.

## Results and Discussion

### High throughput screening and compound identification

**Figure 2:**
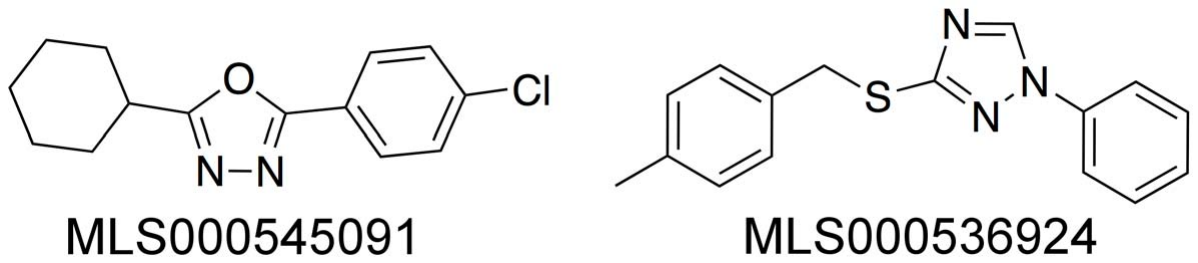
Discovered 15-LOX-2 Selective inhibitors.

To identify novel small molecule inhibitors of 15-LOX-2, we tested a diverse collection of 107,261 compounds arrayed as dilution series (57 µM to 0.73 nM). The 15-LOX-2 qHTS assay utilized the Xylenol Orange colorimetric method for detecting the hydroperoxide reaction products of lipoxygenase as described elsewhere [14], [28]. Complete screening results have been provided in the PubChem public database under Assay Identifier 882. Following the screen and data analysis, screening hits were cherry-picked and retested in the original XO assay; further re-confirmation of inhibitory activity was performed using the orthogonal cuvette-based assay as described in Methods. The compounds shown in Figure 2 were identified as the most promising hits based on their re-confirmed activity and demonstrated selectivity in the cuvette-based lipoxygenase assay.

### Inhibition of Analogues

**Figure 3:**
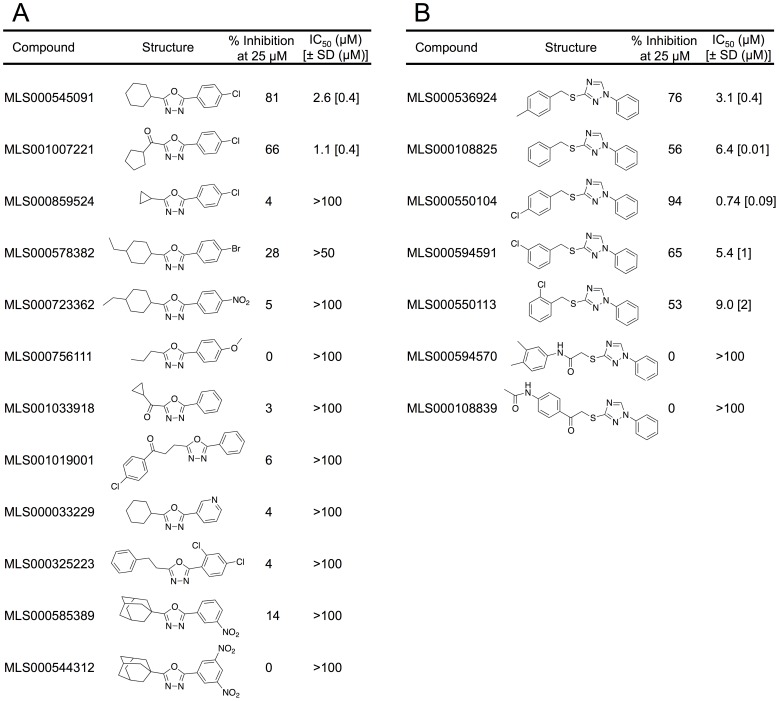
15-LOX-2 IC_50_ values for a few 15-LOX-2 inhibitor analogs, with errors in brackets. (A) MLS000545091 analogs. (B) MLS000536924 analogs. The UV-based manual inhibition data (3 replicates) were fit as described in the Materials and Methods section.

Once the two lead compounds were identified, a limited series of related compounds were identified and screened to investigate structure/activity relationships (Figure 3). Compounds were first screened using a single concentration point at 25 µM. Compounds displaying more than 50% inhibition at 25 µM were then screened at 5 µM, and those displaying greater that 50% inhibition at 5 µM were subjected to full IC_50_ determination, using a minimum of 4 inhibitor concentrations (ranging from 0.5 to 20 µM). Eleven compounds related to MLS000545091 were screened, with varying polarity and steric bulk of both ends of the molecule (Figure 3A). The majority had no activity, but one (MLS001007221) had comparable activity. Six compounds related to MLS000536924 were tested, varying the size and polarity of only one end of the molecule (Figure 3B). Of these six compounds, four had comparable potency to the parent compound, with one being approximately 4-fold more potent (MLS000550104). These data suggest that the parent inhibitors are not promiscuous 15-LOX-2 inhibitors and that it may be possible to improve their potency with further experimentation. We are currently exploring this line of inquiry.

### Compound Selectivity

After identifying two potent 15-LOX-2 inhibitors by high throughput screening, their selectivity was probed using manual screening methods. Compound potency was compared against human LOX isozymes using the UV-Vis assay following hydroperoxide product formation. Both compounds displayed high selectivity for 15-LOX-2 against all enzymes tested (Table 1). MLS000536924 displayed approximately 20-fold selectivity over 15-LOX-1, over 30-fold selectivity compared to 5-LOX and >50-fold over 12-LOX. MLS000545091 showed approximately 20-fold selectivity to the target enzyme over 5-LOX, close to 40-fold over 15-LOX-1, and >50-fold over 12-LOX. These compounds were also tested against COX-1 and COX-2 using an oxygen electrode reaction cell. Both compounds displayed little inhibition against either COX isozyme under the conditions tested.

**Table 1:** Inhibitor IC_50_ values for selected oxygenases.^a^

Compound	15-LOX-2	15-LOX-1	12-LOX	5-LOX	COX-1	COX-2	Redox
MLS000545091	2.6 (0.4)	>100	>100	>50	>50	>100	No
MLS000536924	3.1 (0.4)	>50	>100	>100	>100	>100	No

a The full IC_50_ was performed with 15-LOX-2, but for all other oxygenases, the IC_50_ values were estimated based on one inhibitor point at 20 µM.

All assays were performed in triplicate. The LOX assays were performed with 10 µM AA, while the COX assays were performed with 100 µM AA. The IC_50_ values are in units of micromolar, with error in parentheses.

### Pseudoperoxidase Activity Assay

Many published LOX inhibitors use a redox mechanism to reduce the iron center and inactivate LOX [13]. The possibility of off-target redox chemistry puts redox inhibitors at a disadvantage compared to high affinity competitive inhibitors for therapeutics [29]. The UV-vis pseudoperoxidase assay was used to confirm the absence of redox activity for the current two 15-LOX-2 inhibitors [22]. No degradation of hydroperoxide product was observed at 234 nm, indicating that MLS000536924 and MLS000545091 are not redox active.

### HPLC Inhibitor Modification Assay

To confirm that 15-LOX-2 was incapable of catalyzing chemical transformations of the two 15-LOX-2 inhibitors, HPLC analysis was performed on the compounds after exposure to the enzyme. No significant difference in absorbance spectra or retention time was observed for either of the lead compounds after exposure to 15-LOX-2.

### Steady-State Inhibitor Kinetics

**Figure 4:**
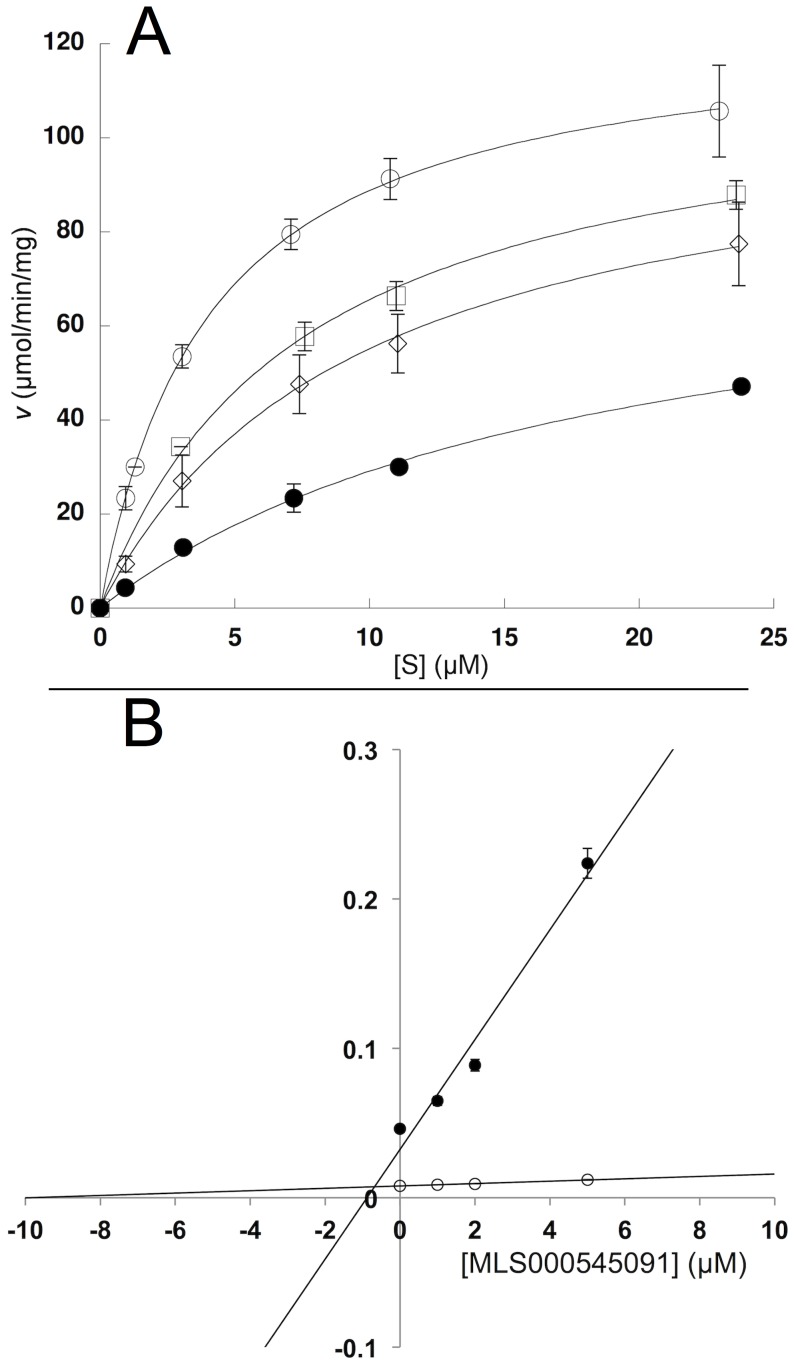
Steady-state kinetics data for the determination of K_i_ and K_i_′ for 15-LOX-2 with MLS000545091. (A) Initial enzymatic rate (µmol/min/mg) versus substrate concentration (µM) at inhibitor concentrations of 0 µM (open circles) 1 µM (open squares) 2 µM (open diamonds) and 5 µM (closed circles) fitted to the Henri–Michaelis–Menten equation to yield *V*_max_ (µmol/min/mg) and *V*_max_/*K*_M_ (mol/min/mg/µM). All measurements were done in triplicate. (B) K_M_/V_max_ replot (closed circles) (units are µM/µmol/min/mg) versus [Inhibitor] (µM), which yielded a K_i_ of 0.9+/−0.4 µM. 1/V_max_ replot (open circles) (units are 1/µmol/min/mg) versus [Inhibitor] (µM), which yielded a K_i_′ of 9.9+/−0.7 µM, indicating weak mixed-type inhibition.

**Figure 5:**
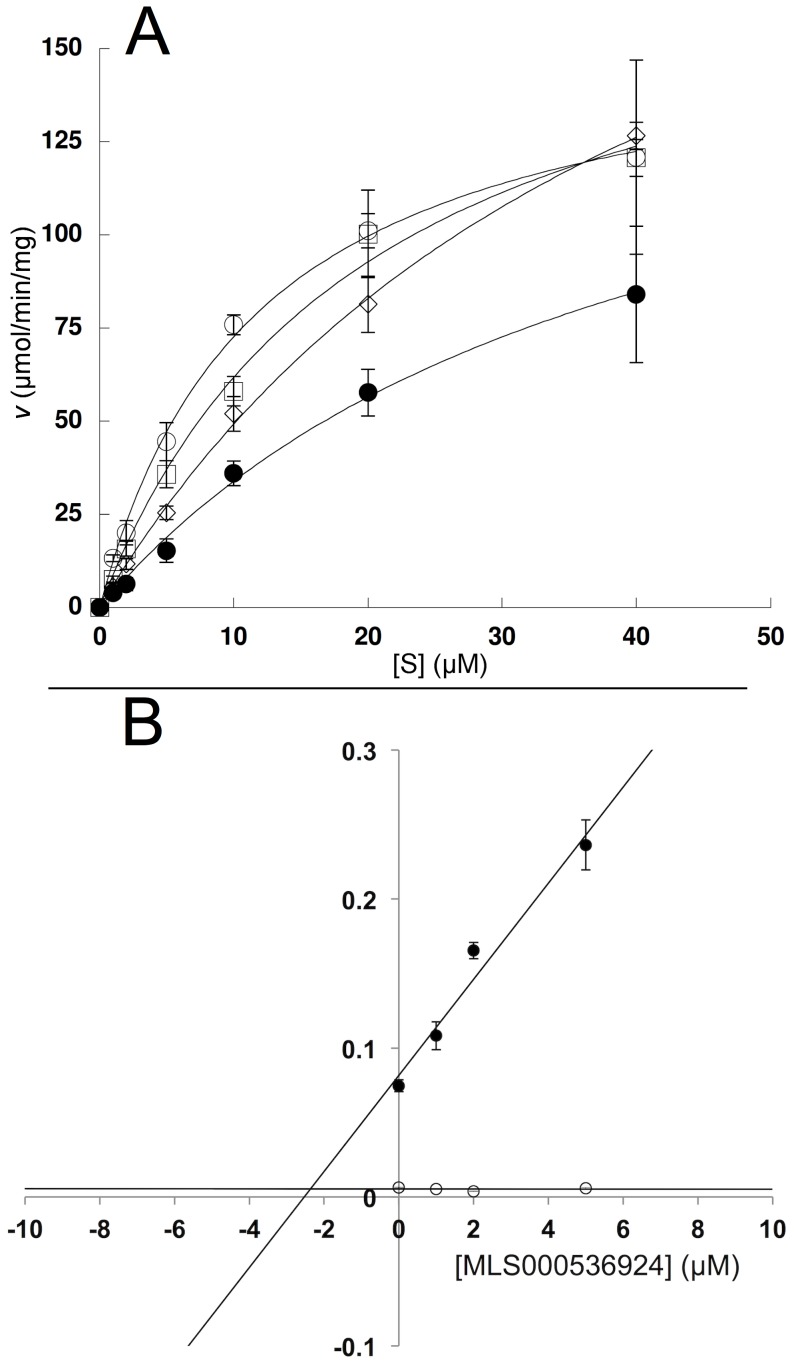
Steady-state kinetics data for the determination of K_i_ for 15-LOX-2 with MLS000536924. (A) Initial enzymatic rate (µmol/min/mg) versus substrate concentration (µM) at inhibitor concentrations of 0 µM (open circles) 1 µM (open squares) 2 µM (open diamonds) and 5 µM (closed circles) fitted to the Henri–Michaelis– Menten equation to yield *V*_max_ (µmol/min/mg) and *V*_max_ */K*_M_ (µmol/min/mg/µM). All measurements were done in triplicate. (B) K_M_/V_max_ replot (closed circles) (units are µM/µmol/min/mg) versus [Inhibitor] (µM), which yielded a K_i_ of 2.5+/−0.5 µM. 1/V_max_ replot (open circles) (units are 1/µmol/min/mg) versus [Inhibitor] (µM), value did not change with increasing inhibitor concentration, indicating competitive inhibition.

The nature of LOX inhibition by the current compounds was further investigated using steady-state kinetics (Table 2). Analysis was performed with MLS000545091 and MLS000536924 by monitoring the formation of 15-HPETE as a function of substrate and inhibitor concentration in the presence of 0.01% Triton X-100. The microscopic rate constants, *V*_max_ (µmol/min/mg) and *V*_max_/*K*_M_ (µmol/min/mg/µM) were determined for MLS000545091 (Figure 4A) and MLS000536924 (Figure 5A) using the Henri–Michaelis–Menten equation. For MLS000545091, the kinetic rate constants were replotted with *K*_M_/*V*_max_ and 1/*V*_max_ versus inhibitor concentration (Figure 4B), yielding K_i_ and K_i_′, respectively. The K_i_ was 0.9+/−0.4 µM and the K_i_′ was 9.9+/−0.7 µM, indicating mixed-type inhibition. The error for K_i_ is relatively high, which could be due to the hydrophobic nature of the inhibitor and its insolubility. For MLS000536924, *K*_M_/*V*_max_ was replotted (Figure 5B) to yield a K_i_ of 2.5+/−0.5 µM. The 1/*V*_max_ was also replotted, but did not change with increasing inhibitor concentration, indicating competitive inhibition. In order to confirm that these compounds do not bind to the allosteric site of 15-LOX-2 appreciably [30], [31], their IC_50_ values were compared using both AA and LA as a substrate, but no significant difference in inhibitor binding was observed (data not shown). This data indicated that allosteric binding does not affect inhibitor potency greatly, which is to be expected given the difference in magnitude between K_i_ and K_i_′ for MLS000545091.

**Table 2:** Inhibitor K_i_ values with AA from Dixon replots.^a^

Compound	K_i_	K_i_′
MLS000545091	0.9 (0.4)	9.9 (0.7)
MLS000536924	2.5 (0.5)	n/a^b^

a All assays were done in triplicate.

The K_i_ was determined from the slope (*K_m_*/*V_max_*) replot at varying inhibitor concentration. The K_i_′ was determined similarly from the y-intercept (1/*V_max_*) replot.

b The y-intercept did not vary with increasing inhibitor, indicating a competitive inhibitor.

The inhibition constants are in units of micromolar, with error in parentheses.

### Computational Docking of Inhibitors to 15-LOX-2

**Figure 6:**
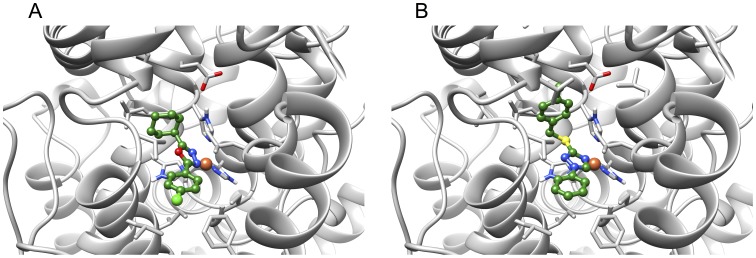
The docking poses of the ligands bound to 15-LOX-2 (A) MLS000545091 and (B) MLS000536924. Carbon atoms of the ligands are shown in green, while the carbon atoms of the protein are shown in grey. The oxygen, nitrogen and hydrogen atoms are shown in red, blue and white colors respectively. The metal ion (Fe^3+^) is shown as an orange sphere. Residues that coordinate to the iron ion are also shown. All water molecules, including the ones that coordinate the metal ion, were deleted prior to docking.

Predicted binding poses of the 15-LOX-2 inhibitors MLS000545091 and MLS000536924 are shown in Figure 6. For both inhibitors, the nitrogen atom of the heterocyclic ring interacts with the iron (distances 2.3 Å and 2.4 Å, respectively) and the flanking hydrophobic rings occupy hydrophobic pockets on either side of the metal ion. Similar to the binding mode of the competitive inhibitor present in the 15-LOX-2 structure [23], both ligands bind in a U-shaped binding mode. In addition to the binding mode, relative docking scores were calculated for all the docked ligands (Table 3), using both the Glide extra precision (XP) docking score and a molecular mechanics based scoring function (MM-GBSA). The Glide XP scoring function has been shown to predict relative binding potencies with reasonable accuracy [27], but in the present work the 15-LOX-2 inhibitors were indistinguishable from the non-binders. The molecular-mechanics energy function based MM-GBSA rescoring [26] more clearly distinguishes between the binders and non-binders.

**Table 3:** The docking scores and relative binding energies of selective 15-LOX-2, 12-LOX and 15-LOX-1 inhibitors docked to the 15-LOX-2 crystal structure.

Compound (Target LOX)	Docking Score (Glide XP)	Relative MM-GBSA Binding Score (kcal/mol)	15-LOX-2 IC_50_ (µM)
MLS000545091 (15-LOX-2)	−7.29	0.0	2.6
MLS000536924 (15-LOX-2)	−7.68	4.3	3.1
ML355 (12-LOX) [16]	−7.36	27.9	>100
ML127 (12-LOX) [15]	−6.27	35.8	>100
ML094 (15-LOX-1) [8]	−9.51	47.0	>100
ML351 (15-LOX-1) [25]	−6.84	67.2	>100

## Conclusions

Using high throughput screening, we have successfully identified two compounds, MLS000545091 and MLS000536924, which selectively inhibit 15-LOX-2 over other oxygenases. These are the first inhibitors of 15-LOX-2 reported in the literature that have low micromolar potency and that do not inhibit the other LOX isozymes. The limited set of derivatives that were investigated demonstrated a structural sensitivity for both compounds, indicating that a more extensive structure/activity relationship study could produce more potent molecules. Interestingly, the degree of similarity in size and shape between the two compounds identified here, MLS000545091 and MLS000536924, may be suggestive of a possible general pharmacophore for successful inhibition of 15-LOX-2. This hypothesis was further supported by computational docking results. For instance, in the predicted binding poses, the oxadiazole or triazole rings interacted with the metal ion and both aromatic rings occupied the hydrophobic channels in a similar manner (U-shaped) to the competitive inhibitor bound in the 15-LOX-2 crystal structure. We are currently probing the structural determinants and cellular activity of these inhibitors, in the hopes of developing effective 15-LOX-2 cellular probes to investigate its role in human disease.
